# U-ResNet, a Novel Network Fusion Method for Image Classification and Segmentation

**DOI:** 10.3390/s25175600

**Published:** 2025-09-08

**Authors:** Wenkai Li, Zhe Gao, Yaqing Song

**Affiliations:** The College of Information, Mechanical and Electrical Engineering, Shanghai Normal University, Shanghai 201418, China; 1000531996@smail.shnu.edu.cn

**Keywords:** image classification, image segmentation, computer vision, ResNet, U-Net

## Abstract

Image classification and segmentation are important tasks in computer vision. ResNet and U-Net are representative networks for image classification and image segmentation, respectively. Although many scholars used to fuse these two networks, most integration focuses on image segmentation with U-Net, overlooking the capabilities of ResNet for image classification. In this paper, we propose a novel U-ResNet structure by combining U-Net’s convolution–deconvolution structure (UBlock) with ResNet’s residual structure (ResBlock) in a parallel manner. This novel parallel structure achieves rapid convergence and high accuracy in image classification and segmentation while also efficiently alleviating the vanishing gradient problem. Specifically, in the UBlock, the pixel-level features of both high- and low-resolution images are extracted and processed. In the ResBlock, a Selected Upsampling (SU) module was introduced to enhance performance on low-resolution datasets, and an improved Efficient Upsampling Convolutional Block (EUCB*) with a Channel Shuffle mechanism was added before the output of the ResBlock to enhance network convergence. Features from both the ResBlock and UBlock were merged for better decision making. This architecture outperformed the state-of-the-art (SOTA) models in both image classification and segmentation tasks on open-source and private datasets. Functions of individual modules were further verified via ablation studies. The superiority of the proposed U-ResNet displays strong feasibility and potential for advanced cross-paradigm tasks in computer vision.

## 1. Introduction

As a core research direction of computer vision (CV), image processing underpins key applications in different fields, such as medical diagnosis [1], autonomous driving [2], industrial inspection [3], etc. Within the domain of image processing, image classification and image segmentation are two fundamental but indispensable tasks. Image classification assigns a single label to an image as a whole [4], while image segmentation assigns labels at the pixel level within an image [5,6]. Convolutional Neural Networks (CNNs) serve as effective solutions for both tasks [7]. Despite significant advancements, persistent challenges in these areas continue to motivate ongoing research.

In the task of image classification, although traditional CNN methods have achieved some success, most of the current state-of-the-art (SOTA) methods mainly focus on extracting global, image-level features for recognition, lacking the ability to capture fine-grained pixel-level details [8]. Because pixels themselves usually contain rich structural information, accurately identifying and utilizing these features enables the model to learn more comprehensive and detailed representations, thereby improving classification accuracy. Moreover, CNN-based image classification methods are often plagued by the vanishing gradient problem during backpropagation derivation, when the network is ultra deep. This phenomenon leads to unstable parameter updates, excessively slow convergence, and overall low learning efficiency. To alleviate the vanishing gradient problem and enable the training of substantially deeper networks, ResNet was introduced [9] and its structure of residual connections led to breakthroughs in image classification.

In contrast, image segmentation demands precise pixel-level feature identification. Networks like U-Net [10] have demonstrated outstanding performance in this task by employing a symmetric encoder–decoder structure with cross-layer connections. This design fuses high-resolution features from shallow layers with semantically rich features from deep layers, mitigating the loss of scale information during encoding and demonstrating strong pixel-level feature extraction capabilities during decoding [11]. Meanwhile, to further enhance the modeling for both short- and long-range contextual information, recent approaches such as Masked Supervised Learning (MaskSup) [12] introduce a framework that leverages random masking during training. Without adding extra parameters, the performance in terms of the average intersection-over-union ratio has been improved by 10%. However, the primary strengths of these networks lie in image segmentation; they are less commonly optimized or evaluated for classification tasks.

Rapid development in image processing requires a single network to handle multiple tasks with sufficient accuracy rates, achieve better computational efficiencies, and solve algorithmic drawbacks such as the vanishing gradient problem. These requirements motivate continuous progression of novel deep learning neural networks. Transformer-focused and CNN-based architectures are two predominant technical approaches.

Along with the development of transformers in the field of NLP, deep learning neural networks have attempted to add self-attention mechanisms at the spatial level and the channel level to capture long-term dependencies for better image processing results. Vision transformer [13] (ViT) validates the effectiveness of the complete transformer structure in computer vision tasks on large-scale training data by dividing images into small patches and introducing one-dimensional positional encoding. GradViT [14] reduces the loss of positional information between image patches in gradient matching by optimizing random noise into natural images during the iterative process. Swin Transformer [15] achieves linear computational complexity with respect to image size by proposing a hierarchical transformer structure that decomposes images into several non-overlapping windows. This decomposition solves the issue faced by ViT methods, where the quadratic computational complexity of self-attention makes it unsuitable for tasks involving high-resolution images. RMT [16] reduces the computational burden during the inference process by extending the temporal decay mechanism of Retentive Network [17] in the field of NLP to the spatial domain and proposing an attention decomposition form that adapts to an explicit spatial prior. These transformer-based structures, however, usually suffer from high training costs and perform poorly in datasets with limited samples. Therefore, networks without attention mechanisms that cause high computational costs are a natural supplementary option.

CNN-based methods, which mainly use convolutional structures for feature extraction, continue to evolve. Non-local neural networks [18], CCNet [19], GCNet [20], Squeeze-and-excitation networks [21], CBAM [22], and ECA-Net [23] are the typical attempts. To mitigate the vanishing gradient problem while increasing image processing efficiencies, more methods have been developed. TDCNDS [24] enhances gradient propagation between convolutional layers by adding auxiliary supervision branches to accelerate convergence speed; GoogleNet [25] achieves multi-scale feature extraction through the Inception module; FCN [26] pioneered the provision of semantic-level image segmentation solutions for CNN architectures; DenseNet [27] strengthens gradient propagation through the construction of densely connected networks; MobileNet [28], EfficientNetv2 [29], and ShuffleNet [30] are dedicated to building lightweight networks; RegNet [31] is an efficient convolutional neural network based on the systematic design rules. These networks have played significant roles in alleviating the gradient vanishing problem or enhancing feature representation, but they mostly focus on optimizing a single task and have not yet explored cross-paradigm fusions.

To resolve multiple tasks simultaneously, ConvNeXt [32] and InceptionNeXt [33] are proposed in sequence. ConvNeXt achieves high-precision classification results at the cost of low FLOPs through large-kernel depth-wise convolution. Drawing on the idea of Inception [25], InceptionNeXt [33] decomposes the large-kernel depth-wise convolution of ConvNeXt along the channel dimension, improving network throughput while maintaining the overall performance. Although ConvNeXt and InceptionNeXt can fuse the image classification and segmentation tasks to some extent, these two models are highly dependent on sophisticated training strategies for ideal results.

The complementary strengths of ResNet and U-Net suggest another reasonable technical approach: integrating these two paradigms together. Prior efforts include ResUNet [34], ResUNet++ [35], and RU-Net [36]. These methods either embed the whole ResNet block within the U-Net encoder or introduce a partial residual connection structure into U-Net [37,38]. Existing studies have also improved segmentation capabilities by designing pyramid dilated convolutions in U-Net [39]. However, most of these approaches focus on image segmentation only, without systematically investigating the potential to excel at both classification and segmentation. Therefore, they cannot multitask very well.

In this paper, following the CNN-based technique approach, we propose a new network architecture, U-ResNet, which is not only able to maintain high accuracy for image classification while enabling fast convergence to mitigate the vanishing gradient problem, but also achieves pixel-level image feature extraction, and therefore is capable of performing superiorly in image segmentation tasks simultaneously.

Our U-ResNet consists of three components: ResBlock (based on ResNet’s residual architecture), UBlock (based on U-Net’s convolutional-deconvolutional framework), and Feature Merge (a component to integrate the features from the above two blocks). In the ResBlock, we leverage the residual connection architecture from ResNet to mitigate the vanishing gradient problem. A Selected Upsampling (SU) module is embedded at the input stage of the ResBlock to upsample low-resolution images, smooth blurred edge features, and facilitate more effective feature extraction for subsequent convolutional layers. An improved Efficient Upsampling Convolutional Block [40] module (EUCB*) is integrated before the end of the ResBlock. The EUCB* enables U-ResNet to achieve faster convergence in classification tasks on high-resolution datasets and thereby greatly improves the learning efficiency. In the UBlock, we adopt the convolutional–deconvolutional structure from U-Net to enable precise pixel-level feature extraction, which is essential for tasks requiring dense prediction (e.g., image segmentation). The UBlock is integrated parallel to the ResBlock, allowing input images to pass through both blocks separately. The final Feature Merge combines the outputs of the ResBlock and UBlock along the channel dimension and makes the final decision for U-ResNet. In this way, our proposed U-ResNet achieves learning capabilities of image classification and segmentation while effectively alleviating difficulties in convergence speed for CNN-based deep learning networks.

To demonstrate the feasibility and advantages of our proposed U-ResNet, we compared it with the current SOTA algorithms in both image classification and segmentation tasks across various typical open-source and private datasets. The benchmark methods in the classification task included (1) RMT [16], a network with a transformer structure; (2) CNN-based networks that can mitigate the vanishing gradient problem, e.g., ResNet [9], MobileNetV2 [41], ShuffleNet [30], and DenseNet [27]; (3) ConvNeXt [32] and InceptionNeXt [33], CNN-based networks for cross-paradigm tasks; and (4) GoogleNet [25], RegNet [31], and EfficientNetV2 [29], networks with modularized CNN structures. In terms of segmentation, the competitors were RMT [16], U-Net [10], and ConvNeXt [32], as well as ResUNet++ [35], which also integrates U-Net and ResNet together.

In all the comparisons, our proposed U-ResNet outperformed the competitors. For image classification, our U-ResNet outperformed all competitors on the two datasets CIFAR-10 [42] and Mini-ImageNet [43], especially the CNN-based multitask methods ConvNeXt and InceptionNeXt. Specifically, U-ResNet demonstrated more trainable characteristics and achieved the fastest convergence during the training process. For image segmentation, we tested U-ResNet in both semantic segmentation tasks with single-class labels (Ice-Seg, which is a private dataset and will be described in Section 3) and multi-class labels (Cityscapes [44] with 19 classes). The proposed U-ResNet performed best in both datasets.

For image classification, we found that on CIFAR-10, U-ResNet enabled near-perfect final accuracy (high *Average* but low *Standard Deviation*, *Top5 Accuracy*: 99.13% ± 0.03%) and fast convergence (on average, 39.2 epochs) on small resolution images (32 × 32). On Mini-ImageNet, U-ResNet still exhibited process stability on higher resolution images (224 × 224) with better final accuracy (*Top1 Accuracy*: 66.78% ± 1.18%) than competitors. For image segmentation, U-ResNet performed especially better in single-class segmentation than in multi-class segmentation. On Ice-Seg, the key metrics (*Precision*, *F1*, *IOU*, *Dice*, *Recall*) from U-ResNet were higher than those on Cityscapes. Overall, the experiments in both tasks demonstrated the superiority of our method.

Meanwhile, we applied 10-fold cross-validation (CV) for both image classification and segmentation to validate the statistical stability of the proposed U-ResNet. We also conducted an ablation study to demonstrate the roles of different modules in our network. Specifically, EUCB*, the resolution for the incremental size of the image data, drives U-ResNet to converge quickly. SU smoothens the edge features of low-resolution images, which is beneficial for the subsequent CNN structure to extract features. The introduction of these two modules is also a major contribution of our work.

Therefore, as a hybrid architecture integrating the advantages of ResNet and U-Net, our U-ResNet exhibits robust comprehensive performance in image classification and segmentation tasks. This model is particularly suitable for classification and semantic segmentation tasks involving medium-to-high-resolution images, e.g., industrial equipment quality inspection and autonomous driving scene recognition, where both accurate object recognition and fine-grained mask prediction are demanded.

However, U-ResNet also suffers from inherent limitations. Due to its complicated structure, U-ResNet exhibits a large parameter volume and high computational cost (in terms of FLOPs), rendering it less suitable for edge devices with constrained computing resources or for applications requiring ultra-low latency. Nonetheless, these limitations highlight potential improvements for our proposed network.

The remainder of this study is organized as follows: Section 2 elaborates on the network architecture and key technical components of U-ResNet in detail. Section 3 presents the experimental configurations and the comparative results. Section 4 concludes this paper and points out potential work for the future.

## 2. Methods

**Figure 1:**
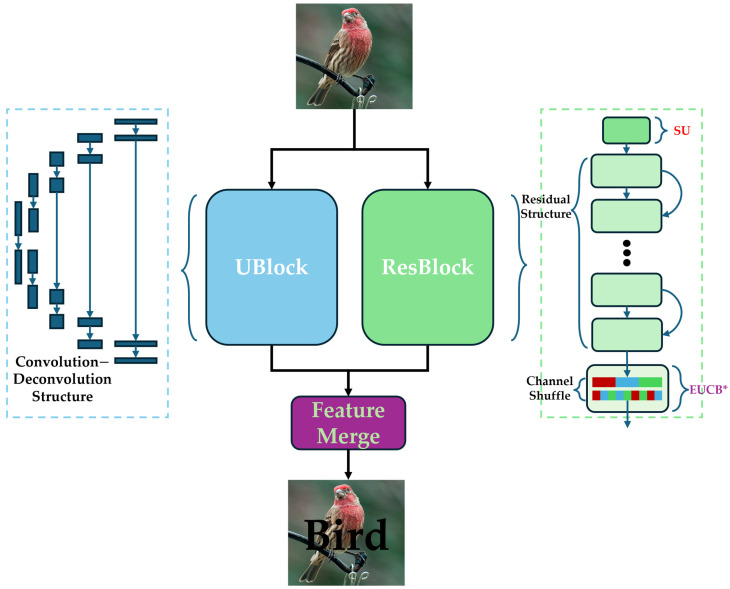
The overall design of U-ResNet.

Displayed in Figure 1, the U-ResNet consists of three major components: ResBlock, UBlock, and Feature Merge. The ResBlock (shown as the light green box in Figure 1) is based on ResNet’s residual architecture and is primarily designed for image classification. The UBlock (as the light blue box in Figure 1) is based on the convolutional-deconvolutional framework of U-Net and focuses on image segmentation. Since ResBlock adopts residual architecture (ResidualBlock) from ResNet, it plays another role in U-ResNet in mitigating the vanishing gradient problem that may cause inconsistent parameter convergence speeds (Please refer to Appendix A for the theoretical analysis). These two blocks are arranged in parallel to enable efficient image processing computations. The Feature Merge module (the purple box in Figure 1) concatenates the features from the ResBlock and UBlock along the channel dimension and generates final outputs for classification and segmentation tasks accordingly.

Two special modules, Selected Upsampling (SU) (highlighted in red in Figure 1) and improved Efficient Upsampling Convolutional Block (EUCB*) (highlighted in purple in Figure 1), were added in the ResBlock for feature extraction at both the pixel level and the level of the whole image. Specifically, the SU module was added at the input stage of the ResBlock to smooth rough edge textures and features for low-resolution images, enabling subsequent ResidualBlocks to better extract relevant features. The EUCB* module was inserted before the fully connected layer of the ResBlock to enable pixel-level feature extraction and representation via calculations of interpolation, channel shuffling, and grouped convolution. Experiments on four datasets demonstrated the innovation, feasibility, and efficiency of these two modules in cross-paradigm tasks of image classification and segmentation. It should be particularly emphasized that the EUCB* module enabled our U-ResNet to achieve faster convergence and higher learning accuracy during the first epoch in classification tasks on high-resolution datasets, thereby improving the overall learning efficiency.

The architecture of our U-ResNet is slightly adapted for image classification and segmentation tasks based on their specific requirements. In the following parts, we will first discuss the major components of the proposed U-ResNet and then introduce the way this network handles classification and segmentation tasks.

### 2.1. Components in U-ResNet

#### 2.1.1. ResBlock

The ResBlock was built on the residual learning structure from ResNet [9]. The “residual” denotes the difference between the observed result and the model’s inference result. Assuming an input x is mapped through a convolutional module and the residual from this module is $F\left(x\right),$ then the mapping result $H\left(x\right)$ is calculated as follows:

(1) $$H\left(x\right)=F\left(x\right)+x.$$

It is generally accepted that simply stacking deep learning layers in a neural network leads to network degradation for image processing [45]. Due to the fast parameter update rate in deep convolutional layers, rapidly changing mappings during training usually distort image features transmitted from the shallow layers, causing network overfitting or degradation. To maintain a better learning capability for a very deep neural network, the residual $F\left(x\right)$ of excessively deep convolutional layers can be set to approximately 0. In this way, the mapping result $H\left(x\right)$ of these deep layers can be expressed as follows:

(2) $$H\left(x\right)\approx x.$$

Therefore, the mapping of the deep layers is an approximate identity mapping, and this mapping prevents network degradation caused by excessively deep layers. At the same time, the skipped layers provide flexible paths for gradients during backpropagation and consequently accelerate network convergence. In this way, the ResBlock is capable of mitigating the vanishing gradient problem in deep neural networks.

**Figure 2:**
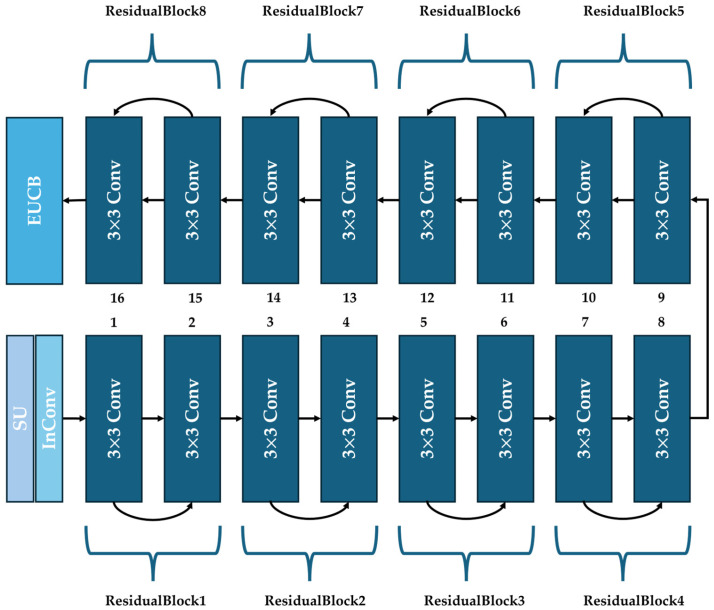
The structure of ResBlock.

Based on the above analysis and inspired by the design of ResNet18 in [8], our ResBlock in U-ResNet is shown in Figure 2, where a residual structure is achieved through shortcut blocks from the $\left(n-1\right)$th to the nth 3 × 3 Conv layers (n is even). This design provides a more convenient propagation path for the gradient, accelerates the convergence speed of parameters in shallow layers, and prevents the degradation of the proposed U-ResNet. We can add or remove ResidualBlocks from this ResBlock as needed to improve the network’s ability to complete different image processing tasks.

#### 2.1.2. Module of Selected Upsampling

**Figure 3:**
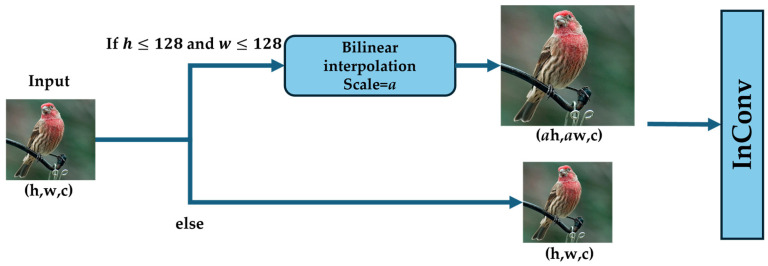
The structure of Selected Upsampling (SU).

To improve feature extraction from low-resolution images (where limited pixels usually constrain the availability of image features) for the proposed U-ResNet structure, we added an SU module (Figure 3) at the input stage of the ResBlock. This module enlarged image size (with length h and width w ≤128 pixels) via the bilinear interpolation method with an integer scaling factor of a. The SU module also smoothed input images by refining coarse textures and features to enhance the image clarity for better recognition. Therefore, the SU module is a preprocessing operation to enable better identification and feature extraction for the subsequent ResidualBlocks structure and can improve the overall performance of our U-ResNet on low-resolution images. The effectiveness of SU was confirmed through ablation study (Section 3.8.1).

#### 2.1.3. EUCB* Module

As the second feature extraction module in ResBlock, EUCB* was modified from the original EUCB [39]. Similarly to the sub-pixel convolution [46] and deconvolutional networks [47], EUCB* is another upsampling method to provide robust structures for pixel-level feature extraction from images. Its design usually combines grouped convolution operations with channel interaction enhancement mechanisms and has shown significant advantages in the tasks of medical image segmentation and semantic segmentation [39].

In the original EUCB module, the processed images from the previous layers are first upsampled via the bilinear interpolation method, then the upsampling results are connected to a 3×3 convolution layer, a Batch Normalization layer, and a ReLU activation function in sequence. Such a design reduces the number of parameters of the convolutional structure but retains computational efficiency. Next, the output of ReLU is shuffled via Channel Shuffle, a sub-module inside the EUCB. During the shuffling conducted by Channel Shuffle, the grouped features from previous layers are rearranged to break feature isolation among channels so that cross-group information can be processed in the following layers. Finally, a layer of 1×1 point convolution is used to compress the channel dimension to the target output size, and the computational complexity is reduced while preserving the image details as much as possible.

**Figure 4:**
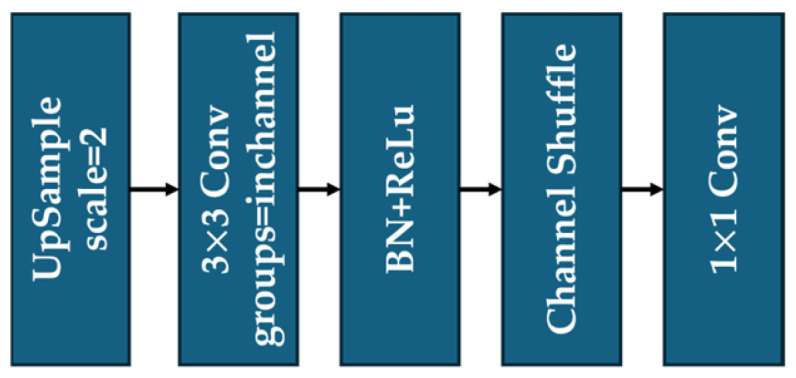
The structure of EUCB.

**Figure 5:**
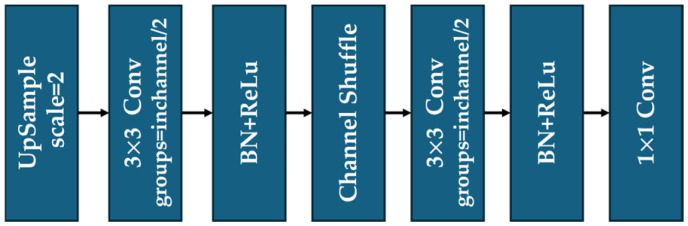
The structure of improved EUCB (EUCB*).

In EUCB*, we halve the group number inside the 3×3 convolutional layer and add another 3×3 convolutional layer with halved groups after the Channel Shuffle operation. This design enables cross-group pixel-level feature interaction across different channel groups within a single EUCB*. As a result, the proposed U-ResNet is equipped with a stronger feature extraction capability in cross-paradigm tasks of image processing. The detailed structure diagrams of the original EUCB and EUCB* are shown in Figure 4 and Figure 5, respectively.

**Figure 6:**
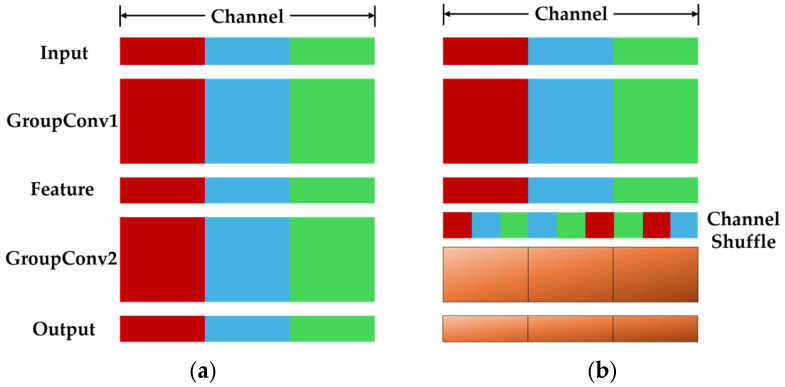
Channel Shuffle diagrams: (**a**) ordinary group convolution without Channel Shuffle, (**b**) grouped convolution with Channel Shuffle.

Channel Shuffle, first proposed in ShuffleNet [30], aims to overcome the side effects caused by group convolution and realize the flow of image information between different groups of channels. Operation without and with Channel Shuffle are compared in Figure 6. From input to output, the same color indicates channels in the same group. In Figure 6b, the network after Channel Shuffle produces more feature extraction paths, which helps the network encode more feature information through parallel processing and therefore significantly improve accuracy.

#### 2.1.4. UBlock

The architecture of the UBlock structure in our U-ResNet is derived from the standard structure of U-Net. While this structure is predominantly used for image segmentation, we adapted it appropriately for image classification tasks.

**Figure 7:**
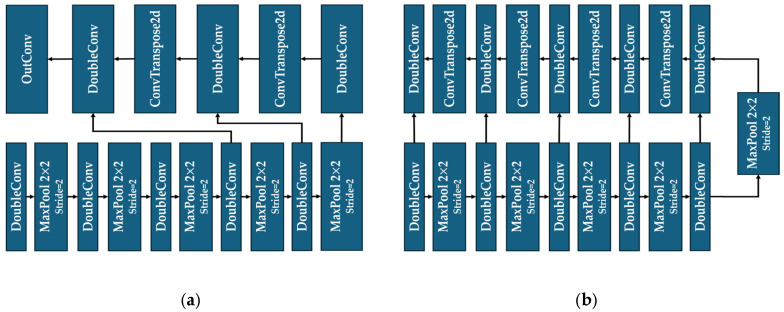
The structure of UBlock (**a**) for image classification and (**b**) for image segmentation.

For image classification, since the feature maps are usually required to be flattened to vectors via fully connected layers, we streamline the UBlock in U-ResNet by reducing its deconvolution and DoubleConv modules. This operation minimizes the spatial size of the output feature maps from UBlock, decreases the number of parameters in the fully connected layer, and accelerates the overall computational efficiencies. Modifying the dimension of the feature maps also ensures that outputs from UBlock align with outputs from ResBlock, guaranteeing subsequent feature fusion operations. For image segmentation, in contrast, the UBlock retains its complete U-shape symmetrical architecture. In this task, the UBlock first follows a pathway of gradual downsampling, convolution, and cross-layer connections, then progressively restores the original image size via deconvolution, thus maintaining its classical U shape framework for pixel-level segmentation accuracy. Details of the UBlock are shown in Figure 7a for image classification and Figure 7b for image segmentation.

#### 2.1.5. Feature Merge

**Figure 8:**
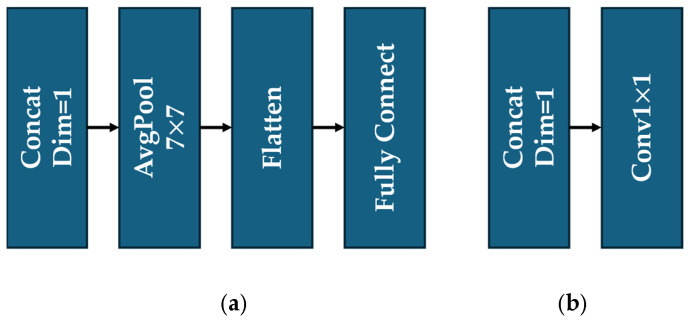
Structures of Feature Merge: (**a**) for image classification, (**b**) for image segmentation.

To effectively fuse features from the ResBlock and UBlock and obtain the corresponding outputs, we used two different Feature Merge modules for image classification and segmentation, respectively. For classification, the output feature maps from the ResBlock and UBlock were first concatenated along the channel dimension, through an average pooling layer, and then flattened into a fully connected layer to generate the classification results. For segmentation, the feature maps were first concatenated along the channel dimension; next, we performed weighted pixel summation across channels for channel-wise feature integration via a point convolution layer, then obtained the segmentation outputs. Structures of these two modules are depicted in Figure 8a,b, respectively.

### 2.2. Architecture for Image Classification

**Figure 9:**
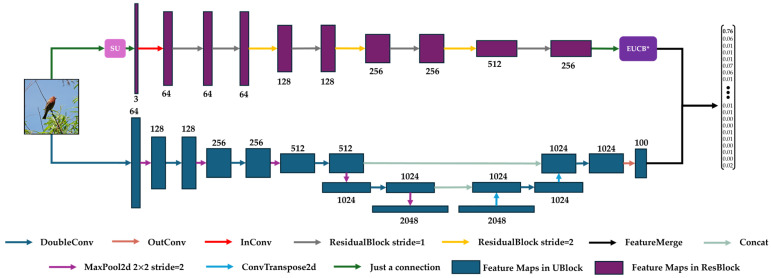
The structural design of U-ResNet for image classification.

The detailed structure of the U-ResNet for the task of image classification is shown in Figure 9. Different colored arrows represent different functional modules or layers and the boxes following next are the corresponding feature map outputs. The SU and EUCB* modules are highlighted in pink and purple, respectively. Values beside the boxes are the number of feature map channels after each functional module or layer. The detailed settings of each critical functional module or layer are listed in Table 1.

For image classification, feature maps are usually required to be flattened to vectors via fully connected layers and it is unnecessary to recover the dimensions of the feature maps to the original size of the input images. Therefore, although the design of the UBlock for classification tasks is based on the standard structure of U-Net, we have reduced the number of its deconvolution layers.

In detail, a DoubleConv module is first used to process the input image into a 64-channel feature map, and then a MaxPool2D layer with size 2 and stride 2 is used to downsample the image, so that the length and width of the image are reduced in half but the number of channels is doubled. In the downsampling stage, this DoubleConv–MaxPool2D process is repeated five times to extract sufficient semantic features of the input image, and then the upsampling operation starts. During upsampling, pixel-level image classification is realized via two pairs of DoubleConv and deconvolution (ConvTranspose2d in Figure 9) modules. Starting from the fourth downsampling step, the feature maps from downsampling are fused to those maps from upsampling via a module of Concate. This fusion enables feature combination from shallow layers and deep layers, strengthening gradient conduction and enriching mapping ability for image semantics. Finally, an OutConv module is applied to compress the number of feature channels from the UBlock to the same number as that from the ResBlock for Feature Merge.

In ResBlock, the stride of each ResidualBlock is set to 1 or 2. During image processing, a stride value of 1 does not change the size of the feature map or the number of channels, while a stride value of 2 compresses the width and height of the image to half but doubles its number of channels. After 8 ResidualBlocks, the feature map extracted from the input image enters the EUCB* module for pixel-level feature extraction to obtain the output of the ResBlock. The size of outputs from both the ResBlock and UBlock are controlled to 28 × 28 to simplify the subsequent computations. Next, the outputs from the two blocks are concatenated in Feature Merge to make the final classification decision.

### 2.3. Architecture for Image Segmentation

**Figure 10:**
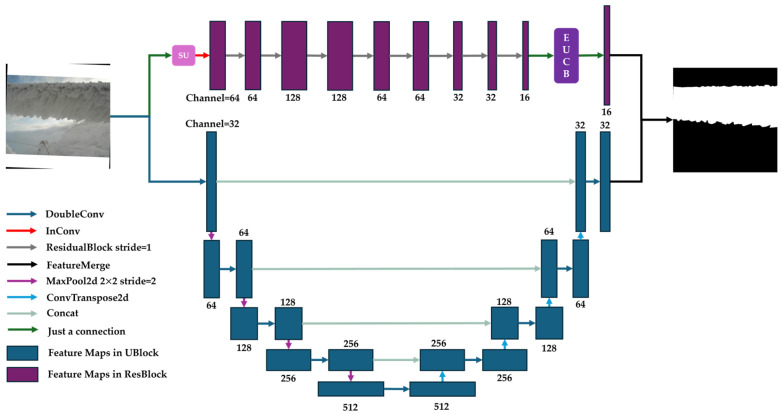
The structural design of U-ResNet for image segmentation.

Figure 10 shows the detailed structure of U-ResNet for image segmentation. Different colored arrows represent different functional modules or layers while the following boxes are the feature mapping outputs. Modules of SU and EUCB* are highlighted in pink and purple, respectively. The values beside the boxes are the number of feature map channels after each functional module or layer. The detailed settings of each critical functional module or layer are listed in Table 2.

For image segmentation, our design of UBlock is basically the same as the design for image classification, except that the number of instances of downsampling is reduced from 5 to 4 and the number of instances of upsampling is increased from 2 to 4. In segmentation, from the first step of the UBlock, the feature maps from downsampling are fused to the maps from upsampling. This is different from the UBlock designed for image classification.

In ResBlock, the stride of each ResidualBlock is kept as 1 to keep the size of the feature map constant when the map flows through the network. In order to reduce the amount of calculation for subsequent ResidualBlocks and to accelerate the speed of model inferences, other parameters are kept consistent with those for image classification. After 8 ResidualBlocks, the feature maps are upsampled back to the size of the original image via EUCB*.

Once the outputs from both the ResBlock and UBlock are obtained, they are concatenated into Feature Merge for the final segmentation mask.

## 3. Experiment

In this section, the capabilities of the proposed U-ResNet for image classification and segmentation were demonstrated via experiments on open-sourceand private datasets. Ablation studies were conducted to check the effectiveness and efficiency of the special modules introduced into our network. Statistical significance was analyzed via 10-fold CV to verify the expectation and stability of our method given randomness. The code and models are publicly available at https://github.com/Nanlingxq/U-ResNet.

### 3.1. Experiment Environment

All the experiments were conducted on an NVIDIA 3090 GPU with a 12-core CPU and 48GB RAM. PyTorch 2.5.1, CUDA 12.1, and cuDNN 9 were the software stacks.

### 3.2. Experiment Datasets

Four datasets were applied in the experiments, two for image classification and two for segmentation.

CIFAR-10 [42], a typical open-source dataset for image classification, contains 60,000 samples with an image size of 32 × 32, covering 10 image classes. This dataset was randomly partitioned into 50,000 samples for training and 10,000 samples for testing.

Mini-ImageNet [43], a subset of the renowned open-source ImageNet [48] classification benchmark dataset, includes 100 classes with 600 images per class. This dataset was randomly split into training and testing sets with a ratio of 4:1. Each image was resized to 224 × 224 RGB resolution during data preprocessing.

Cityscapes [44], a public semantic segmentation dataset, mainly covers urban streets scenes for urban scene recognition studies. This dataset contains 5000 high-resolution images with fine annotations (resolution of 1024 × 2048) and an additional 20,000 images with coarse annotations. The 5000 fine-annotated images were used in our experiment.

Ice-Seg (please refer the details in Appendix B), a private dataset for power grid icing segmentation, contains 1591 manually labeled power grid icing images. A total of 1467 images were randomly selected for training and 124 for testing. The resolution of each image is 640 × 640.

### 3.3. Benchmark Algorithm

To thoroughly validate the performance of our method as well as to demonstrate the advantages of the special modules, the benchmark methods were chosen to cover as much and diverse of the SOTA algorithms which use deep neural network as possible.

- For Image Classification

Twelve benchmark methods were considered: (1) the CNN-based networks GoogleNet (using Inception), MobileNetV2 (based on depth-wise Convolution), RegNet (using reverse residual blocks), ShuffleNet (introducing Channel Shuffle), DenseNet (using dense connections), and EfficientNetV2 (using efficient architectures), as well as three variants of ResNet for the vanishing gradient problem, ResNet18, ResNet34, and ResNet50; (2) ConvNeXt and InceptionNeXt, CNN-based networks for cross-paradigm tasks and also equipped with a convolutional structure inspired by the design principles of Swin Transformer; (3) RMT, a network with a transformer structure.

- For Image Segmentation

The benchmark methods were (1) U-Net, a classical segmentation network; (2) RMT, a transformer-structure-based method; (3) ResUNet++, another network which integrates ResNet features into a U-Net structure; (4) ConvNeX, a method for cross-paradigm tasks equipped with a convolutional structure inspired by Swin Transformer.

### 3.4. Experiment Metrics

- For Image Classification

*Top1 Accuracy* (*Top1* in short) and *Top5 Accuracy* (*Top5*) were the two metrics to evaluate the classification accuracy of the model. Top1 represents the percentage of the data whose true class label is the predicted label from the algorithm, while Top5 represents the percentage of the data whose true class label belongs to the top five predicted labels from the algorithm. These two metrics can be calculated as follows:

(3) $$Top1\;=\frac{T_{1}}{Total},$$

and

(4) $$Top5\;=\frac{T_{5}}{Total},$$

where Total represents the total number of data, $T_{1}$ is the number of data points whose true class label is the predicted label from the algorithm, and $T_{5}$ is the number of data points whose true class label belongs to the top five predicted labels from the algorithm.

- For Image Segmentation

Six metrics, *Accuracy*, *Precision*, *Recall*, *F1 Score*, *IoU*, and *Dice Coefficient*, were applied for image segmentation. Specifically, *Accuracy* measures the proportion of correctly classified pixels and is calculated as follows:

(5) $$Accuracy=\frac{TP+TN}{TP+TN+FP+FN},$$

where *TP* represents the correctly classified foreground pixels, *TN* represents the correctly classified background pixels, *FP* is the background pixels incorrectly classified as foreground pixels, and *FN* is the foreground pixels incorrectly classified as background pixels.

*Precision* is the ratio of correctly identified foreground pixels to all pixels predicted as foreground and is represented as follows:

(6) $$Precision=\frac{TP}{TP+FP}.$$

*Recall* represents the proportion of foreground pixels that are correctly detected:

(7) $$Recall=\frac{TP}{TP+FN}.$$

The *F1 Score* is the harmonic mean of *Precision* and *Recall*, balancing their trade-off:

(8) $$F1=2\times \frac{Precision\times Recall}{Precision+Recall}.$$

*IoU* (Intersection over Union) quantifies the overlap between predicted and ground truth segmentation areas, with values ranging from 0 to 1 (a value close to 1 means that the number of incorrectly classified pixels is close to 0, indicating a better performance).

(9) $$IoU=\frac{TP}{TP+FP+FN}.$$

The *Dice Coefficient* assesses the similarity of pixel-level overlap, which is particularly useful for imbalanced datasets (e.g., small-target segmentation).

(10) $$Dice\;Coefficient=\frac{2\times TP}{2\times TP+FP+FN}.$$

While *Accuracy*, *Precision*, and *Recall* are single-indicator metrics, *F1*, *IoU*, and *Dice Coefficient* are composite metrics that provide more comprehensive evaluations of algorithm performance. By leveraging these metrics, we aimed to thoroughly assess the architectural features and learning outcomes of U-ResNet for segmentation tasks.

### 3.5. Experiment Setting

- For Image Classification

In CIFAR-10, we set the batch size to 64 and applied 100 epochs for training. An SGD optimizer was used with an initial learning rate of 0.01. The learning rate was reduced by factor of 0.1 if the training accuracy plateaued (variance < 3% from average) for 10 consecutive epochs. All the images were normalized.

In Mini-ImageNet, we also set the batch size to 64, applied 100 epochs for training, and adopted SGD as the optimizer. The initial learning rate is set to 0.1, then reduced to 0.01 at epochs 41–80, and further reduced to 0.001 at epochs 81–100. All the images were normalized and random flipping was applied.

- For Image Segmentation

In both datasets of Cityscapes and Ice-Seg, we had a batch size of 8, 50 training epochs, Adam optimizer, and an initial learning rate of 1 × 10^−4^ and normalized the images.

### 3.6. Classification Results

#### 3.6.1. Value of the Results

The experimental results for all 12 benchmark methods as well as the proposed U-ResNet on the datasets CIFAR-10 and Mini-ImageNet are listed in Table 3 and Table 4, respectively. Besides *Top1* and *Top5*, ‘Epoch of Convergence’ was also considered to compare the training speed and convergence efficiency among the algorithms. ‘FLOPs’ and ‘#Param (Number of Parameters)’ were used to check the computational complexity. The results from U-ResNet and the best results from the competitors are highlighted in bold.

#### 3.6.2. Analysis of the Results

- Classification Accuracy

Displayed in Table 3 and Table 4, on dataset CIFAR-10, the proposed U-ResNet achieved the best *Top1* (86.94%) and *Top5* (99.24%) classification accuracies. Since the size of images in CIFAR-10 is only 32 × 32, this result is able to demonstrate the structural advantages of our proposed method in extracting accurate classification features from low-resolution images.

Given the dataset Mini-ImageNet, the proposed U-ResNet also achieved the best results for the metric of *Top5* (89.75%). Although EfficientNetV2 and DenseNet obtained slightly better results in *Top1* (70.88% and 70.74%), performance from our method still ranked number three, only 0.54% and 0.4% worse than EfficientNetV2 and DenseNet. With both *Top1* and *Top5* accuracies in consideration, our method can be regarded as one of the best, if not the only one, among all the 13 methods. Since the type of images in Mini-ImageNet covers 100 different classes, this result displays the capability of our U-ResNet to recognize diversified images with high accuracy.

- Convergence Efficiency

The ‘Epochs of Convergence’ for all the 13 methods were documented to check the training speed and convergence efficiency. ‘Epochs of Convergence’ represent the number of epochs after which the performance of an algorithm stops improving significantly during training. A smaller number of ‘Epochs of Convergence’ indicates a faster training speed or more efficient performance improvement with parameter updates. For a deep learning neural network, a smaller number of ‘Epochs of Convergence’ usually implies the network will not suffer from the vanishing gradient problem. Therefore, this metric was also used to analyze how the parallel integration of the ResBlock and UBlock might affect the vanishing gradient problem for our U-ResNet.

Given Mini-ImageNet, our U-ResNet converged fastest among all the methods (the same epochs as ShuffleNet). Given CIFAR-10, although slightly slower than EfficientNetV2 and RegNet (39 epochs vs. 36 epochs), our method can still be regarded as one of the best. This result, consequently, indicates that our U-ResNet can rapidly learn effective image features in early training stages with strong learning efficiency from diversified images, and is capable of mitigating the vanishing gradient problem.

- Result Explanations

The experimental results can be first explained by the proposed parallel structure of the ResBlock and UBlock in our U-ResNet. This structure integrates the residual structure in the ResBlock and the cross-layer connections in the UBlock and therefore can update the network parameters efficiently and effectively to facilitate gradient propagation and mitigate gradient vanishing for a very deep neural network. Meanwhile, the EUCB* module in the ResBlock and the shallow deconvolution structure in the UBlock enhance the pixel-level feature extraction, which enables our U-ResNet to capture key features faster and achieve convergence in fewer iterations, thus improving the training efficiency.

- Computational Complexity

We have to acknowledge that the proposed U-ResNet has a higher computational complexity, as displayed by higher values of FLOPs and more #Param in both Table 3 and Table 4. In the classification task, we consider this complexity to be mainly from the UBlock. A detailed analysis will be presented in Section 3.8.1.

#### 3.6.3. Comparison to Other Cross-Paradigm Algorithms for Image Classification

ConvNeXt and InceptionNeXt were considered in the experiments to compare the performance on cross-paradigm tasks for our proposed U-ResNet.

Although the accuracy was slightly superior to that of ConvNeXt and InceptionNeXt (in CIFAR-10, 86.94% > 85.12% and 78.88% for *Top1* and 99.24% > 97.41% and 94.98 for *Top5*; in Mini-ImageNet, 70.34% > 69.61% and 69.35% for *Top1* and 89.75% > 84.68% and 87.10% for *Top5*), our U-ResNet displayed a significantly faster convergence speed (in CIFAR-10, 39 < 79 and 46; in Mini-ImageNet, 41 < 88 and 79). These results indicate our U-ResNet can obtain a near-perfect balance between classification performance and convergence efficiency compared to other networks for cross-paradigm tasks.

A more rigid advantage of our method in comparison to ConvNeXt and InceptionNeXt lies in the low dependence on training strategies (please check the detailed training environments and programming settings for ConvNeXt and InceptionNeXt in Appendix C). The performance of ConvNeXt and InceptionNeXt greatly relies on sophisticated training techniques (e.g., smooth decay of learning rate) and adaptive optimizers (e.g., Adam). In contrast, our U-ResNet can still stably converge to a relatively excellent performance when using simplified training strategies such as traditional SGD (without momentum). This reflects that the parameter optimization landscape of U-ResNet is smoother, with a smaller gap between local minima and potentially better solutions, making the gradient descent process less affected by fluctuations in training strategies and thus reducing the need for complex hyperparameter scheduling.

#### 3.6.4. Statistical Analysis for Image Classification

To account for potential randomness during dataset partition and to validate the generalization of our method, the proposed U-ResNet underwent 10-Fold CV on selected datasets of CIFAR-10 and Mini-ImageNet. Values of the mean and standard deviation of the *Top1* and *Top5* accuracies, as well as the ‘Epoch of Convergence’ after 10-Fold CV, are listed in Table 5. All parameters were kept the same during validation.

The results in Table 5 indicate the cross validation successfully captured the randomness during dataset partition. One can first tell that the means of the *Top1* and *Top5* accuracies after 10-Fold CV was slightly lower than the one-time values (in CIFAR-10, 85.15% < 86.94% for *Top1* and 99.13% < 99.24% for *Top5*; in Mini-ImageNet, 66.78% < 70.34% for *Top1* and 87.88% < 89.75 for *Top5*). This indicates that datasets with small batches are more sensitive to training sample selection, and random dataset partition can lead to imbalanced class labels, resulting in declining model performance. One can also find that our method is stable over the selected datasets, since the standard deviation of the classification accuracies after 10-Fold CV was extremely low (in CIFAR-10, 0.57 for *Top1* and 0.03 for *Top5*; in Mini-ImageNet, 1.18 for *Top1* and 1.01 for *Top5*). Therefore, we can conclude uncertainties will not easily interrupt our U-ResNet for image classification.

About the training speed and convergence efficiency, given a 99% (z=2.58, 99% confidence level corresponds to the z value) success rate, the confidence interval (*CI*) of the number of ‘Epoch of Convergence’ after 10-Fold CV can be obtained via the following:

(11) $$CI={\bar{N}}_{epoch}\pm 2.58\frac{{std}_{epoch}}{\sqrt{10}},$$

where ${\bar{N}}_{epoch}$ is the average number of ‘Epochs of Convergence’ and ${std}_{epoch}$ is the standard deviation of the ‘Epochs of Convergence’. Therefore, based on the values in Table 5, the *CI*s for ‘Epoch of Convergence’ in the CIFAR-10 dataset are $\left[37.0,\;41.3\right]$ and in Mini-ImageNet are $\left[42.5,\;46.3\right]$, almost covering the values of 39 and 41 from U-ResNet in a one-time experiment. Furthermore, by comparing the *CI*s to the values in Table 3 and Table 4, the upper bounds (the worst cases given 99% confidence level) of the two *CI*s, 41.3 and 46.3, can still rank among the top values in the one-time experiments.

In summary, 10-Fold CV demonstrated the generalizability of our method and strengthened our conclusion that the proposed U-ResNet performs effectively and efficiently in tasks of image classification.

### 3.7. Segmentation Results

The experimental results on the Ice-Seg and Cityscapes datasets are shown in Table 6 and Table 7, respectively. The results indicate that U-ResNet has significant advantages in pixel-level semantic segmentation tasks. The results from U-ResNet and the best results from the competitors are highlighted in bold.

#### 3.7.1. Results and Analysis

On the Ice-Seg dataset, U-ResNet exhibited notable strengths. Against U-Net and ResUNet++, our method achieved higher scores across all six metrics, demonstrating superior overall performance in segmentation for ice-covered transmission lines. When compared to RMT, U-ResNet outperformed RMT in *Accuracy* (94.40% > 94.12%), *Precision* (88.67% > 85.77%), *F1 Score* (86.22% > 85.94%), *IoU* (75.78% > 75.35%), and *Dice Coefficient* (86.22% > 85.94%), though it lagged slightly in *Recall* (83.90% < 86.11%). Compared to ConvNeXt, U-ResNet surpassed it in all metrics, including *Accuracy* (94.40% > 89.17%), *Precision* (88.67% > 70.53%), *Recall* (83.90% > 82.67%), and key segmentation metrics like *IoU* (75.78% > 61.45%). These results confirm the capability and robustness of U-ResNet in extracting pixel-level features, particularly in capturing ice-covered features on power transmission lines, leveraging fused features from the parallelized UBlock and ResBlock.

On Cityscapes, our method maintained its advantages and outperformed all competitors across all six metrics. This demonstrates U-ResNet’s strong ability to accurately classify urban scene pixels, minimize missegmentation rates, comprehensively cover target regions, and reduce missed detection rates in complex urban semantic segmentation tasks.

It is worth noting that the complexity of our method for image segmentation is at a moderate level. The computational complexity for image segmentation are from both the UBlock and the ResBlock. We will discuss this in Section 3.8.2 in detail.

#### 3.7.2. Explanations via Segmented Images

**Figure 11:**
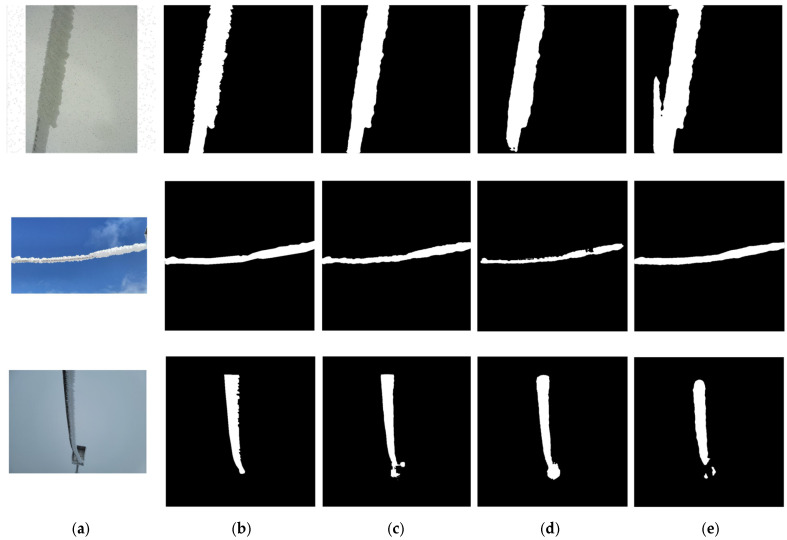
The segmented images from Ice-Seg, (**a**) the input real image, (**b**) ground truth—true segmented images, (**c**) the results from the proposed U-ResNet, (**d**) the results from ResUnet++, (**e**) the results from RMT.

The experimental results can also be explained via the segmented images. Figure 11 illustrates the segmented ice-covered high-voltage line images for all methods in the Ice-Seg dataset. As it shows, the predicted mask via U-ResNet (Figure 11c) closely matches the true mask (Figure 11b). In complex backgrounds, such as the junctions between wires and insulators on transmission lines, U-ResNet demonstrates a rigid capability for detail-preserving discriminations, enabling the detection of subtle image changes or nuanced differences between different image regions.

In contrast, the segmentation results from ResUnet++ (Figure 11d) and RMT (Figure 11e) exhibit obvious shortcomings. ResUnet++ produced blurred edge boundaries (lower *Accuracy*) which highlighted the effectiveness and advantages from the SU module and EUCB* modules that we added to the ResBlock in U-ResNet. These two modules can fully leverage multi-scale image information to accurately define target edges and thereby capture image edge features and details. Conversely, the RMT algorithm suffered from missed detections, where areas that should have been identified as transmission lines were not correctly segmented (lower *Precision*). This indicates the cross-layer connections designed in U-ResNet’s initial downsampling stage in the UBlock can effectively capture the majority of target pixels, leading to a better segmentation performance.

#### 3.7.3. Comparison to Other Cross-Paradigm Algorithms for Image Segmentation

We compare the proposed U-ResNet to ConvNeXt in the task of image segmentation. As performed in image classification, ConvNeXt has harsh requirements for the training environment and training strategy in image segmentation and may return extreme results if the segmentation head is not properly selected (please check details in Appendix C). In contrast, U-ResNet only requires traditional SGD and simple training strategies to enable segmentation metrics to converge stably. This indicates that our U-ResNet is easier to train, and therefore easier for practice, in tasks of image segmentation.

#### 3.7.4. Statistical Analysis for Image Segmentation

10-Fold CV on the Ice-Seg dataset was conducted to validate the generalization of the proposed U-ResNet given randomness during semantic segmentation. The means, standard deviations, and confidence intervals (given 99% confidence level) of the six metrics of *Accuracy*, *Precision*, *Recall*, *F1 Score*, *IoU*, and *Dice Coefficient* are listed in Table 8.

For image segmentation, we also adopted the coefficient of variation to verify the stability of the metrics during cross validation. The coefficient of variation is calculated as follows:

(12) $$\hat{C_{v}}=\frac{Std}{Average}\times 100\%,$$

where Std and Average are the standard deviation and the mean of the metrics, respectively. Displayed in Table 8, after 10-Fold CV, the $\hat{C_{v}}$ of all the six metrics on Ice-Seg were less than 4%, indicating strong segmentation stability from U-ResNet. The lower bounds of the CIs of the six metrics were almost all better than the best results of the corresponding metrics from the one-time segmentation experiment (*Accuracy* 95.43% > 94.13%, *Precision* 93.19% > 92.85%, *Recall* 90.50% > 86.11%, *F1 Score* 91.87% > 85.94%, *IoU* 85.70% > 75.35%, *Dice Coefficient* 91.91% > 85.94%). This indicates that our cross validation provided more comprehensive evaluations through multiple dataset partitions, neutralizing drawbacks from a biased sample distribution in a one-time experiment. Briefly, our U-ResNet displays outstanding generalizability for image segmentation tasks.

### 3.8. Ablation Study

To better investigate the performance from individual modules in our U-ResNet, ablation studies were conducted for image classification and segmentation, respectively.

#### 3.8.1. Ablation Study in Classification

The effects of individual functional modules on classification accuracies (*Top1* and *Top5*) as well as training speed and convergence efficiency (‘Epoch of Convergence’) are presented in Table 9, where ‘E’ represents the EUCB* module and ‘S’ represents the Selected Upsampling module. Since the UBlock is based on the convolutional–deconvolutional framework of U-Net, a classical network for semantic segmentation task, the UBlock is the third functional module for image classification and is denoted as ‘U’.

Our proposed network with all functional modules is denoted as U-ResNet. The network without module ‘S’ is U-ResNet-1, and that without module ‘E’ is U-ResNet-2, without module ‘U’ is U-ResNet-3. U-ResNet-4 (equivalent to ResNet18) is the network without all functional modules and is the baseline in the ablation study for image classification. Since it is not necessary to upsample the high-resolution images (224 × 224 in Mini-ImageNet), the SU module does not work in this scenario and therefore the same results were obtained from U-ResNet and U-ResNet-1 for the Mini-ImageNet dataset in Table 9. The results from the best networks are highlighted in bold and it is the same for all the ablation studies in this section.

- Analysis of Individual Modules

First is the overall performance. From Table 9, U-ResNet-2, which is without the functional module ‘E’, returned the best classification accuracy (both *Top1* and *Top5*) for the selected datasets CIFAR-10 and Mini-ImageNet. U-ResNet and U-ResNet-1 on Mini-ImageNet and U-ResNet-2 on CIFAR-10 return the best training speed and convergence efficiency (the best number of ‘Epochs of Convergence’).

Then is the EUCB* module. Comparing U-ResNet and U-ResNet-2, although EUCB* may slightly reduce the classification accuracy (U-ResNet vs. U-ResNet2, on CIFAR-10, 1.07% decreases for *Top1* and 0.28% decreases for *Top5*; on Mini-ImageNet, 0.77% decreases for *Top1* and 0.42% decreases for *Top5*), this module can notably accelerate the training convergency for high-resolution images in Mini-ImageNet (reduces the ‘Epoch of Convergence’ from 54 to 41, more than 20%). Therefore, we can conclude EUCB* enables U-ResNet to achieve a faster convergence speed to classify high-resolution images and significantly mitigates the vanishing gradient problem.

Next is the SU. Checking the value of ‘Epoch of Convergence’ and comparing the accuracies between U-ResNet and U-ResNet-1 for the dataset CIFAR-10, the proposed U-ResNet with SU accelerated the training speed by reducing the converged epoch number from 48 to 39 and increased the *Top1* accuracy from 86.47%. to 86.94%. Therefore, the ablation study guarantees the SU module is able to smooth rough edge textures and features for low-resolution images, making them clearer for convolutional layers to identify and hence extract them for image discrimination.

Finally, we consider the UBlock. Comparing the performance between U-ResNet and U-ResNet-3 (CIFAR-10 *Top1*: 87.03% vs. 83.15%; Mini-ImageNet *Top1*: 70.34% vs. 63.47%), the inclusion of the UBlock significantly boosted the classification accuracy. These results demonstrate that the UBlock can enhance the model’s classification ability across different resolution datasets by enabling pixel-level feature extraction.

- The Scale Parameter in SU

Besides the functional comparison among individual modules, critical parameters were also optimized via the ablation study. In Table 10, we have displayed the effectiveness on classification accuracy from the upsampling scale factor a in the SU module. Since it is not necessary to adopt SU in Mini-ImageNet, only the results from CIFAR-10 are listed. In Table 10, with the scale a increasing from 1 to 4, the *Top1* accuracy first rises and then declines, reaching its maximum when a is 3. This result also indicates the optimal operation to upsample low-resolution images for the best feature extraction results, and this is the value we used for all the experiments in this paper.

- Further Analysis of EUCB*

To better understand the role of EUCB*, we conducted another ablation study by processing four image resolutions of 224 × 224, 112 × 112, 64 × 64, and 32 × 32 in Mini-ImageNet in turn through the EUCB* module. Table 11 displays the results, and two phenomena can be found: (1) for low-resolution images, EUCB* puts more weight on accuracy than efficiency; (2) for high-resolution images, EUCB* can return significantly better efficiency while slightly sacrificing accuracy.

Specifically, given an image resolution of 32 × 32, compared to the network without EUCB*, the U-ResNet with EUCB* had better classification accuracy (*Top1* 51.99% > 50.36% and *Top5* 77.45% > 76.50%) and worse training speed and convergence efficiency (‘Epochs of Convergence’ 54 > 41). Increasing the resolution to 64 × 64, the U-ResNet with EUCB* started to return a slightly worse classification accuracy (*Top1* 57.81% < 60.47% and *Top5* 82.09% < 83.92%) and an almost equal training speed and convergence efficiency (‘Epochs of Convergence’ 49 ≈ 45). Further increasing the resolution to 112 × 112 and 224 × 224, the U-ResNet with EUCB* could obtain a significantly better training speed and convergence efficiency (‘Epochs of Convergence’ 43 < 55 and 41 < 54) by slightly sacrificing classification accuracy (*Top5* 86.71% < 87.97% and 89.75% < 90.17%; *Top1* 65.23% < 67.30% and 70.34% < 71.11%).

This phenomenon can be explained as follows: (1) When the image resolution is low, e.g., 32 × 32 or 64 × 64, an image usually has little information (features) to explore. Thereby the EUCB* module may introduce redundant parameter updates, causing there to be more steps to achieve stable convergency. (2) When the image resolution increases to 112 × 112 and 224 × 224, more detailed information (features) needs to be recognized and the EUCB* module can help our U-ResNet focus on key features more efficiently with reduced ineffective explorations, thereby shortening the convergence path.

Consequently, the ablation study on EUCB* reflects the adaptability of this module to the complexity of the input images. It also provides a training strategy for our proposed U-ResNet: for low-resolution images, the EUCB* module can be bypassed for a rapid convergence speed; for high-resolution images, EUCB* can be added to balance the classification accuracy with the training cost.

In summary, the ablation study for image classification displays that all three modules, SU, EUCB* and UBlock, work together to provide outstanding classification results. For algorithm convergence, all three modules, especially the EUCB* module, help to mitigate the vanishing gradient problem. For classification accuracy, the SU module is necessary for low-resolution images while the UBlock provides the ability, across images with different resolutions, to achieve accurate feature extractions. EUCB* may slightly sacrifice the classification accuracy to efficiency, especially for high-resolution images. Practical instructions on using the EUCB* module are also provided.

- Analysis of Computational Complexity in Classification

From Table 9, it is evident that, in image classification tasks, the computational complexities can be primarily attributed to the UBlock. Although the UBlock in our U-ResNet has been simplified, it retains the multiple convolution–deconvolution modules and skip connections from the original U-Net. These components introduce a substantial number of parameters and significant computational overhead.

#### 3.8.2. Ablation Study in Segmentation

An ablation study for image segmentation was also conducted and the effects of the individual functional modules for the metrics of *Accuracy*, *Precision*, *Recall*, *F1 Score*, *IOU*, and *Dice Coefficient* are presented in Table 12. Since EUCB* plays a significant role in our U-ResNet, this module was studied and denoted as ‘E’. For image segmentation, the UBlock is the essential backbone. Therefore, ResBlock, the complementary part of the UBlock, was evaluated in this ablation study and is denoted as ‘R’.

As in the study on image classification, the U-ResNet with all functional modules is defined as ‘U-ResNet’. The network without module ‘E’ is U-ResNet-2 and the network without module ‘R’ is U-ResNet-3 (similar to U-Net). Since module ‘E’ is a part of module ‘R’, when module ‘R’ is removed from the U-ResNet, module ‘E’ is also removed.

Let us consider the Ice-Seg dataset first. EUCB* primarily enhances the target localization during image segmentation. Removing EUCB* (U-ResNet-2) caused a slight drop in *Precision* (from 88.67% to 88.30%) but a significant reduction in *Recall* (from 83.90% to 82.41%) and *IoU* (from 75.78% to 74.30%), highlighting the role of this module to recognize integrated targets. The ResBlock mainly enhances segmentation stability and its removal (U-ResNet-3) led to drastic drops in all metrics. These results also indirectly demonstrate that the residual connections in the ResBlock are effective, as they alleviate the vanishing gradient problem, preserve deep image features, and prevent missed detection of unconventional targets like thin ice layers.

On Cityscapes, the role of EUCB* was primarily reflected in suppressing false positives and enhancing the precision of target segmentation. The removal of EUCB* (U-ResNet-2) led to a drop in Precision (from 80.70% to 78.41%), significant reductions in the F1 Score (from 78.78% to 76.36%) and IoU (from 73.83% to 70.29%), and a relatively stable Accuracy (only a slight decrease from 98.24% to 98.05%). This indicates that EUCB* effectively reduces background misclassification (false positives) through multi-scale feature interaction. Regarding the ResBlock, its removal (U-ResNet-3, equivalent to a simplified U-Net structure) resulted in a mixed trend: Recall increased slightly (from 79.32% to 80.88%), Precision dropped from 80.70% to 76.86%, the F1 Score decreased from 78.78% to 77.34%, and IoU fell from 73.83% to 70.20%. This suggests that the residual connections in the ResBlock play a critical role in stabilizing feature propagation and enhancing the result consistency for segmentations. Although the simplified U-Net captured more foreground pixels (higher Recall) with less model complexity, it lacked the complementary features fused from the ResBlock, leading to a compromised overall performance.

From Table 12, it is evident that the UBlock constitutes one of the primary sources of computational complexity in image segmentation, as the values of the FLOPs and parameters (#Param) do not change significantly upon removal of either the EUCB* or the ResBlock. As noted earlier, the UBlock comprises multiple convolution–deconvolution operations and cross-layer feature fusion through skip connections. These components considerably enhance the model’s capacity while imposing a substantial computational burden, particularly when processing high-resolution images. Additionally, the ResBlock itself contributes a notable number of parameters due to its stride configurations, convolutional layers, and residual connections. Moreover, the group convolution and channel shuffling within the EUCB* further increase the number of FLOPs. In summary, both the UBlock and the ResBlock are major contributors to the computational cost for image segmentation.

**Figure 12:**
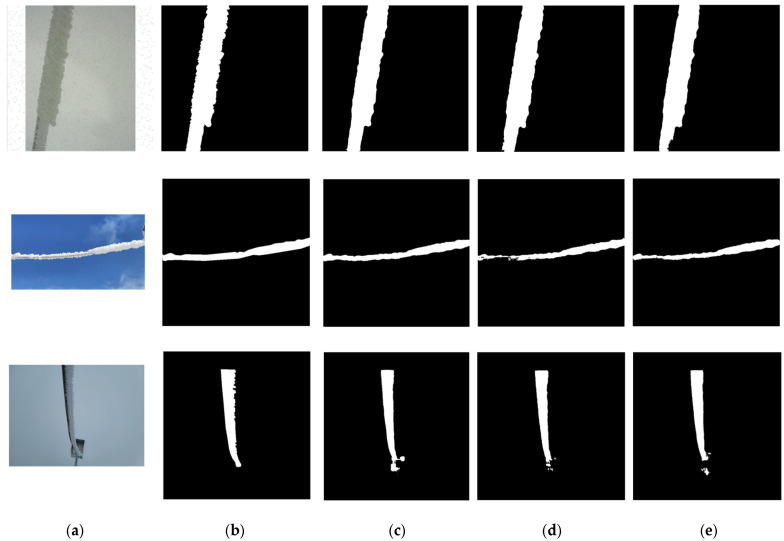
The ablation image segmentation results for the Ice-Seg dataset: (**a**) the input real image, (**b**) ground truth—the true segmented images, (**c**) the results from the proposed U-ResNet, (**d**) the results from U-ResNet-2, (**e**) the results from U-ResNet-3 (similar to U-Net).

The semantic segmentation results for ice-covered high-voltage line images from the three models are displayed in Figure 12. One can tell that the segmentation results from U-ResNet are most consistent with the actual situation. They exhibit clear contours of target objects, fully displayed details, and distinct foregrounds and backgrounds. These images are also perfectly consistent with the excellent performance displayed in Table 10.

From the ablation study for segmentation, we can conclude that the ResBlock stabilizes the performance and therefore ensures segmentation robustness while EUCB* improves segmentation accuracy and provides segmentation adaptability per task requirements. In summary, the synergistic integration of EUCB*, the ResBlock, and the backbone of UBlock, equips our U-ResNet with enhanced adaptability and superior performance on complex image segmentation tasks.

## 4. Conclusions

In this paper, we propose a highly generalizable, accurate dual-task neural network, U-ResNet, for classical image processing tasks (classification and segmentation). U-ResNet integrates the ResBlock (adopting ResNet’s residual structure) and the UBlock (based on U-Net’s convolutional-deconvolutional framework) as parallel backbones. The modules Selected Upsampling (SU) and improved EUCB (EUCB*) are added to the input and end stages of the ResBlock, respectively. Feature maps from the ResBlock and UBlock are merged in a Feature Merge module to make final image processing decisions.

Such an architecture equips U-ResNet with strong pixel-level feature extraction capabilities for low- and high-resolution images in both tasks. Specifically, ResBlock’s residual structures and UBlock’s cross-layer connections alleviate gradient vanishing in backpropagation. SU smooths rough edges in low-resolution images, facilitating feature extraction and identification; EUCB* and the shallow deconvolutions in the UBlock boost pixel-level feature extraction, capturing key features faster to improve training results.

The performance of the U-ResNet on both image classification and segmentation tasks was evaluated. For classification, the open-source datasets CIFAR-10 (low-resolution) and Mini-ImageNet (high-resolution) were used, and U-ResNet outperformed comparison models in classification accuracy and convergence efficiency. For segmentation, the open-source Cityscapes and private Ice-Seg datasets were used to cover diverse resolutions and semantic masks, and U-ResNet surpassed competitors with higher segmentation accuracy and lower prediction false positives.

Statistical analysis via 10-fold CV was conducted for both tasks to examine performance stability under non-negligible randomness, which confirmed the strong generalizability of our method. Ablation studies were carried out to verify the effectiveness and efficiency of each module, highlighting the necessity and practicality of the U-ResNet architecture. The optimal SU scale factor was also identified. These results confirm U-ResNet’s ability to handle both tasks for diverse images in complex practical scenarios.

We have to acknowledge that U-ResNet has a relatively high computational complexity. For classification, the complexity is mainly from the UBlock; for segmentation, it comes from both the UBlock and ResBlock. Further verification/optimization is recommended for scenarios with limited computing resources or extreme resolutions.

In future work, our U-ResNet can be improved in three aspects. First, we can improve the real-time performance with more effective computing strategies or design novel convolution structures for better computational efficiency (e.g., optimizing the convolution–deconvolution and cross-layer connections in the UBlock to reduce values of FLOPs and #Param while maintaining high accuracy). Second, we can introduce channel attention mechanisms to facilitate a deeper understanding of the feature fusion between the outputs of the ResBlock and UBlock. Third, we can adopt resolution-independent neural operators to handle diversified image resolutions.

## Figures and Tables

**Table 1:** The specific design parameters of U-ResNet for image classification.

Functional Module	Number of Module	Output Size (h×w) from Each Module	Layer
DoubleConv	8	(1) 224 × 224 (5) 14 × 14 (2) 112 × 112 (6) 7 × 7 (3) 56×56 (7) 14 × 14 (4) 28 × 28 (8) 2 × 28	$\left[\begin{array}{c} 3\times 3\;Conv\\ BN+ReLu\\ 3\times 3\;Conv\\ BN+ReLu \end{array}\right]$
OutConv	1	28 × 28	$\left[\begin{array}{c} BN+ReLu\\ 1\times 1\;Conv \end{array}\right]$
InConv	1	112 × 112	$\left[\begin{array}{c} Selected\;Upsample\\ 7\times 7\;Conv\\ BN+ReLu \end{array}\right]$
ResidualBlock Stride = 1	5	(1) 112 × 112 (4) 28 × 28 (2) 112 × 112 (5) 14 × 14 (3) 56 × 56	$\left[\begin{array}{c} 3\times 3\;Conv\\ BN+ReLu\\ 3\times 3\;Conv\\ BN\\ Shortcut \end{array}\right]$
ResidualBlock Stride = 2	3	(1) 56 × 56 (3) 14 × 14 (2) 28 × 28	$\left[\begin{array}{c} 3\times 3\;Conv\\ BN+ReLu\\ 3\times 3\;Conv\\ BN\\ Shortcut \end{array}\right]$
Shortcut	1	/	$\left[\begin{array}{c} 1\times 1\;Conv\\ BN \end{array}\right]$
EUCB*	1	28 × 28	$\begin{array}{c} Upsample\\ \left[\begin{array}{c} 3\times 3\;Group\;Conv\\ BN+ReLu \end{array}\right]\\ Channel\;Shuffle\\ \left[\begin{array}{c} 3\times 3\;Group\;Conv\\ BN+ReLu \end{array}\right]\\ 1\times 1\;Conv \end{array}$
FeatureMerge	1	1 × 100 vector	$\left[\begin{array}{c} Concat\;dim=1\\ Flatten\\ Fully\;Connection \end{array}\right]$

**Table 2:** The specific design parameters of U-ResNet for image segmentation.

Functional Module	Number of Modules	Output Size (h×w) of Each Module	Layer
DoubleConv	9	(1) 640 × 640 (5) 40 × 40 (2) 320 × 320 (6) 80 × 80 (3) 160 × 160 (7) 160 × 160 (4) 80 × 80 (8) 320 × 320 (9) 640 × 640	$\left[\begin{array}{c} 3\times 3\;Conv\\ BN+ReLu\\ 3\times 3\;Conv\\ BN+ReLu \end{array}\right]$
OutConv	1	640 × 640	$\left[\begin{array}{c} BN+ReLu\\ 1\times 1\;Conv \end{array}\right]$
InConv	1	320 × 320	$\left[\begin{array}{c} Selected\;Upsample\\ 7\times 7\;Conv\\ BN+ReLu \end{array}\right]$
ResidualBlock	1	320 × 320	$\left[\begin{array}{c} 3\times 3\;Conv\\ BN+ReLu\\ 3\times 3\;Conv\\ BN\\ Shortcut \end{array}\right]$
Shortcut	1	**/**	$\left[\begin{array}{c} 1\times 1\;Conv\\ BN \end{array}\right]$
EUCB*	1	640 × 640	$\begin{array}{c} Upsample\\ \left[\begin{array}{c} 3\times 3\;Group\;Conv\\ BN+ReLu \end{array}\right]\\ Channel\;Shuffle\\ \left[\begin{array}{c} 3\times 3\;Group\;Conv\\ BN+ReLu \end{array}\right]\\ 1\times 1\;Conv \end{array}$
Feature Merge	1	640 × 640	$\left[\begin{array}{c} Concat\\ OutConv \end{array}\right]$

**Table 3:** The image classification experimental results for the dataset CIFAR-10.

Model	Top1	Top5	Epoch of Convergence	FLOPs	#Param
ResNet18	80.26	98.86	38	0.03 G	0.18 M
ResNet34	82.65	98.85	39	0.06 G	0.51 M
ResNet50	82.03	99.07	53	0.07 G	0.54 M
GoogleNet	75.93	97.22	65	0.03 G	5.98 M
MobileNetV2	70.25	96.83	63	0.01 G	3.22 M
RegNet	63.83	95.43	**36**	0.07 G	14.30 M
ShuffleNet	82.64	99.09	50	0.01 G	0.85 M
DenseNet	83.44	99.00	72	0.16 G	26.49 M
RMT	75.11	97.42	40	0.20 G	53.12 M
EfficientNetV2	57.36	92.96	**36**	0.12 G	52.87 M
ConvNeXt	85.12	97.41	79	0.31 G	87.61 M
InceptionNeXt	78.88	94.98	46	0.31 G	83.61 M
Ours	**86.94**	**99.24**	**39**	**1.04 G**	**42.61 M**

**Table 4:** The image classification experimental results for the dataset Mini-ImageNet.

Model	Top1	Top5	Epoch of Convergence	FLOPs	#Param
ResNet18	64.70	87.70	48	1.82 G	11.38 M
ResNet34	64.13	85.82	48	3.68 G	21.49 M
ResNet50	62.25	84.71	51	4.13 G	24.33 M
GoogleNet	69.22	89.08	73	1.58 G	6.08 M
MobileNetV2	64.23	86.18	73	0.59 G	3.31 M
RegNet	57.18	82.95	44	3.22 G	14.39 M
ShuffleNet	62.19	84.90	41	0.17 G	1.03 M
DenseNet	**70.74**	89.67	62	7.84 G	26.69 M
RMT	60.65	84.04	56	18.92 G	94.42 M
EfficientNetV2	**70.88**	89.38	45	5.44 G	52.87 M
ConvNeXt	69.61	84.68	88	15.35 G	87.61 M
InceptionNeXt	69.35	87.10	79	14.86 G	83.89 M
Ours	**70.34**	**89.75**	**41**	**17.40** **G**	**138.24** **M**

**Table 5:** The 10-Fold CV results on the CIFAR-10 and Mini-ImageNet datasets.

Dataset: CIFAR-10 Model: U-ResNet			
Metrics	Top1	Top5	Epoch of Convergence
Mean	85.15	99.13	39.2
Standard Deviation	0.57	0.03	2.68
**Dataset: Mini-ImageNet Model: U-ResNet**			
**Metrics**	**Top1**	**Top5**	**Epoch of Convergence**
Mean	66.78	87.88	44.1
Standard Deviation	1.18	1.01	2.28

**Table 6:** The semantic segmentation results of Ice-Seg dataset.

Dataset: Ice-Seg								
Model	Accuracy	Precision	Recall	F1 Score	IOU	Dice Coefficient	FLOPs	#Param
Ours	**94.40**	88.67	83.90	**86.22**	**75.78**	**86.22**	437.75 G	31.96 M
ResUnet++	91.72	85.32	72.90	78.62	64.77	78.62	98.84 G	4.06 M
U-Net	93.09	**91.49**	73.78	81.68	69.04	81.68	434.10 G	31.93 M
RMT	94.12	85.77	**86.11**	85.94	75.35	85.94	359.39 G	70.75 M
ConvNeXt	89.17	70.53	82.67	76.12	61.45	76.12	515.48 G	123.11 M

**Table 7:** Semantic segmentation results of Cityscapes dataset.

Dataset: Cityscapes								
Model	Accuracy	Precision	Recall	F1 Score	IOU	Dice Coefficient	FLOPs	#Param
Ours	**98.24**	**80.70**	**79.32**	**78.78**	**73.83**	**78.78**	561.08 G	31.96 M
ResUNet++	97.20	75.22	60.87	63.29	57.12	63.29	126.66 G	4.06 M
U-Net	97.42	78.09	75.81	75.81	68.91	75.81	438.64 G	31.04 M
RMT	97.12	71.84	66.52	67.41	60.68	67.41	550.66 G	99.40 M
ConvNeXt	97.56	74.23	70.42	70.62	64.22	70.62	1221.00 G	103.73 M

**Table 8:** The results of the 10-Fold CV on the Ice-Seg dataset.

Dataset: Ice-Seg Model:U-ResNet						
Metrics	Accuracy	Precision	Recall	F1	IOU	Dice Coefficient
Mean	96.59	93.19	90.50	91.87	85.70	91.91
Standard Deviation	1.42	2.04	2.58	2.31	3.04	2.30
Coefficient of Variation	1.48%	2.19%	2.85%	2.51%	3.55%	2.50%
Confidence Interval	95.43, 97.75	91.53, 94.85	88.39, 92.61	89.99, 93.75	83.22, 88.18	90.04, 93.78

**Table 9:** The ablation study results of U-ResNet for image classification.

Dataset: CIFAR-10								
Model	E	U	S	Top1	Top5	FLOPs	#Param	Epoch of Convergence
U-ResNet	√	√	√	86.94	99.24	1.04 G	42.61 M	39
U-ResNet-1	√	√	×	86.47	99.26	1.03 G	42.60 M	48
U-ResNet-2	**×**	**√**	**√**	**88.01**	**99.52**	**1.68 G**	**42.09 M**	**27**
U-ResNet-3	√	×	√	83.15	99.04	0.61 G	11.50 M	40
U-ResNet-4	×	×	×	80.26	98.86	0.03 G	0.18 M	38
**Dataset: Mini-ImageNet**								
**Model**	**E**	**U**	**S**	**Top1**	**Top5**	**FLOPs**	**#Param**	**Epoch of Convergence**
U-ResNet	√	√	√	70.34	89.75	17.40 G	138.24 M	**41**
U-ResNet-1	√	√	×	70.34	89.75	17.40 G	138.24 M	**41**
U-ResNet-2	**×**	**√**	**√**	**71.11**	**90.17**	**17.29 G**	**132.65 M**	54
U-ResNet-3	√	×	√	63.47	85.73	7.04 G	11.72 M	51
U-ResNet-4	×	×	×	64.70	87.70	1.82 G	11.38 M	44

‘√’ represents the network with this module and ‘×’ represents without this module.

**Table 10:** The *Top1 Accuracy* from different upsampling scales for CIFAR-10.

Model	Scale a	Top1 Accuracy
U-ResNet	1	81.44
U-ResNet	2	85.33
U-ResNet	**3**	**86.94**
U-ResNet	4	86.73

**Table 11:** The ablation study on EUCB* for Mini-ImageNet with different image resolutions.

W and H	E	Top1	Top5	Epochs of Convergence
32	√	51.99	77.45	54
32	×	50.36	76.50	41
64	√	57.81	82.09	49
64	×	60.47	83.92	45
112	√	65.23	86.71	43
112	×	67.30	87.97	55
224	√	70.34	89.75	41
224	×	71.11	90.17	54

‘√’ represents the network with this module and ‘×’ represents without this module.

**Table 12:** The ablation study results for image segmentation.

Dataset: Ice-Seg										
Model	E	R	Accuracy	Precision	Recall	F1 Score	IOU	Dice Coefficient	FLOPs	#Param
U-ResNet	**√**	**√**	**94.40**	**88.67**	**83.90**	**86.22**	**75.78**	**86.22**	437.75 G	31.96 M
U-ResNet-2	×	√	94.02	88.30	82.41	85.25	74.30	85.25	434.10 G	31.93 M
U-ResNet-3	×	×	92.16	83.84	77.55	80.57	67.47	80.57	342.22 G	31.09 M
**Dataset: Cityscapes**										
**Model**	**E**	**R**	**Accuracy**	**Precision**	**Recall**	**F1 Score**	**IOU**	**Dice** **Coefficient**	**FLOPs**	**#Param**
U-ResNet	**√**	**√**	**98.24**	**80.70**	79.32	**78.78**	**73.83**	**78.78**	561.08 G	31.96 M
U-ResNet-2	×	√	98.05	78.41	76.31	76.36	70.29	76.36	556.40 G	31.93 M
U-ResNet-3	×	×	98.00	76.86	**80.88**	77.34	70.20	77.34	438.64 G	31.04 M

‘√’ represents the network with this module and ‘×’ represents without this module.

**Figure A1:**
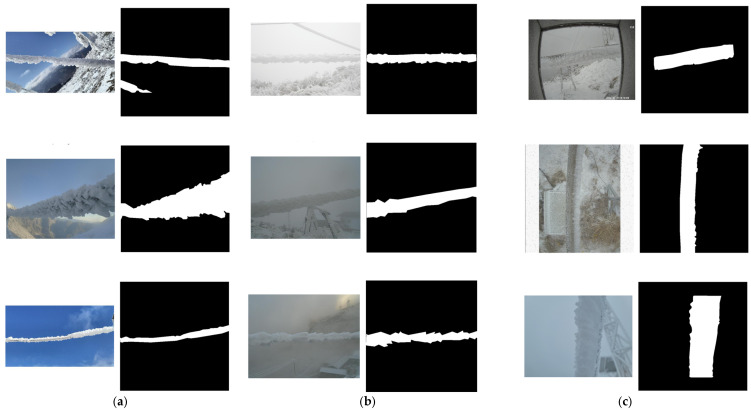
Visualization examples of images and annotated masks from IceSeg: (**a**) images taken under good weather conditions, (**b**) images taken under harsh weather conditions, (**c**) images with other equipment in the background.

## Data Availability

The Ice-Seg dataset applied in this paper is a private dataset within our project team. And the image classification datasets are all open-source datasets. The open-source URLs are shown as follows. Mini-ImageNet: https://www.kaggle.com/datasets/arjunashok33/miniimagenet. CIFAR-10: https://www.cs.toronto.edu/~kriz/cifar.html. Cityscapes: https://www.cityscapes-dataset.com/.

## Abbreviations

The following abbreviations are used in this manuscript:

EUCB	Efficient Upsampling Convolutional Block
EUCB*	Improved Efficient Upsampling Convolutional Block
CNNs	Convolutional Neural Networks
SOTA	State-Of-The-Art
SU	Selected Upsampling
CV	Cross Validation

## Appendix A. The Vanishing Gradient Problem in U-ResNet

As it was discussed in Section 2.1 and was demonstrated via the experimental tests in Section 3.6 and Section 3.7, as well as the ablation studies in Section 3.8, the parallel integration of the ResBlock and UBlock in our U-ResNet can effectively mitigate the vanishing gradient problem. We provide a theoretical analysis in Appendix A.

In U-ResNet, given the input x, the outputs from the ResBlock are denoted as $F_{r}(x;\theta_{r})$ and from the UBlock are $F_{u}(x;\theta_{u})$, where $\theta_{r}$ and $\theta_{u}$ are parameters in the ResBlock and UBlock, and $F_{r}$ and $F_{u}$ are the operations in these two blocks, respectively. The feature fusion performed in the module of Feature Merge can be expressed as follows:

(A1) $$F_{merge}\left(x\right)=C\left(F_{r}\left(x;\theta_{r}\right),F_{u}\left(x;\theta_{u}\right)\right),\;$$

where C denotes the channel concatenation in Feature Merge. Assume the loss function of our U-ResNet is L, then the gradients to the loss function can be derived as follows:

(A2) $$\frac{\partial L}{\partial \theta_{r}}=\frac{\partial L}{\partial C}\times \left(\frac{\partial C}{\partial F_{r}}\times \frac{\partial F_{r}}{\partial \theta_{r}}+\frac{\partial C}{\partial F_{u}}\times \frac{\partial F_{r}}{\partial F_{u}}\right),$$

(A3) $$\frac{\partial L}{\partial \theta_{u}}=\frac{\partial L}{\partial C}\times \left(\frac{\partial C}{\partial F_{r}}\times \frac{\partial F_{r}}{\partial \theta_{u}}+\frac{\partial C}{\partial F_{u}}\times \frac{\partial F_{u}}{\partial \theta_{u}}\right),$$

and the Jacobian matrices are given by the following:

(A4) $$\frac{\partial C}{\partial F_{r}}=\left[\begin{array}{c} I\\ 0 \end{array}\right],\;\;\;\;\;\;\frac{\partial C}{\partial F_{u}}=\left[\begin{array}{c} 0\\ I \end{array}\right],\;$$

where I is the identity matrix. Equation (A4) implies that the two gradients of $\frac{\partial L}{\partial \theta_{r}}$ and $\frac{\partial L}{\partial \theta_{u}}$ are mutually independent. Since the ResBlock and UBlock do not share parameters in the proposed U-ResNet, we can get the following:

(A5) $$\frac{\partial F_{r}}{\partial \theta_{u}}=0,\;\;\;\;\;\;\frac{\partial F_{u}}{\partial \theta_{r}}=0.\;$$

Finally, given the learning rate η, the parameters in our U-ResNet are updated via the following:

(A6) $$\theta_{r}^{\left(t+1\right)}=\theta_{r}^{\left(t\right)}-\eta \times \frac{\partial L}{\partial \theta_{r}^{\left(t\right)}};$$

(A7) $$\theta_{u}^{\left(t+1\right)}=\theta_{u}^{\left(t\right)}-\eta \times \frac{\partial L}{\partial \theta_{u}^{\left(t\right)}};$$

and the two updating processes are completely independent. Therefore, we can conclude that the backpropagation process in the ResBlock will not affect the process in the UBlock and vice versa. We can further conclude that our U-ResNet mitigates the vanishing gradient problem for the ResBlock and the UBlock independently.

In the ResBlock, by virtue of the residual connections, the structure can avoid the convergence and vanishing gradient problems, which was discussed in Section 2.1. Benefiting from the design of the convolutional–deconvolutional structure, the length of the UBlock is usually not very deep and therefore this structure is less prone to the vanishing gradient problem. In this parallel and independent way, our proposed U-ResNet mitigates the vanishing gradient problem.

In summary, the parallel arrangement of the ResBlock and UBlock in the proposed U-ResNet alleviates the vanishing gradient problem while incorporating outstanding pixel-level feature extraction capabilities for complex image processing scenarios.

## Appendix B. The Ice-Seg Dataset

The Ice-Seg dataset is a private dataset for single-class semantic segmentation. In Ice-Seg, 1591 images were taken to capture icing conditions on high-voltage cables. All the images are in JPG format with a uniform resolution of 640 × 640. We purchased this dataset and annotated the images manually on the online platform Roboflow [49].

We categorized the images into three groups based on the weather conditions and background characteristics: (a) images captured under good weather conditions (e.g., sunny days); (b) images captured under harsh weather conditions; (c) images containing other equipment in the background. Examples of these categories with their annotated masks are shown in Figure A1. In the training set, there were 115 samples from class (a), 1252 from class (b), and 100 from class (c). In the testing set, there were 11 samples from class (a), 99 from class (b), and 14 from class (c). The training set consisted of a total of 1467 samples, while the test set contained a total of 124 samples.

Challenges exist and requirements vary to correctly segment images under these categories. The core challenge with images taken on sunny days is the high likelihood of false positives caused by visually similar objects, mainly manifested in the confusion between ice and clouds/bright skies, reflections/highlights, and backgrounds. The edges of clouds, reflections on cable surfaces, or backgrounds like snowfields are similar to ice in terms of color and brightness, making them prone to being misjudged. The key to solving this lies in enabling the model to learn spatial location and contextual features, clarifying that ice must be attached to the cable itself, and distinguishing through textural differences (ice is more irregular, clouds are fluffier, and reflections are smoother).

The core challenge with images captured under harsh weather conditions is the low contrast, noise, and blurred targets, which easily lead to false negatives and inaccurate contour recognition. Given low visibility, the contrast between ice and the background is low; noise such as falling snow or raindrops may either mask the ice signal or be misjudged as ice. Additionally, the contour of ice is too blurred to distinguish clearly, resulting in an extremely high risk of false negatives. Addressing these issues requires enhancing the model’s robustness and improving capabilities in abstract feature extraction, long-range dependency modeling, and weak edge detection. Meanwhile, the design of the loss function should specifically penalize false negatives.

The core challenge with images containing equipment in the background is false positives caused by foreground–background confusion and interference from similar structures. The geometric shapes of equipment like high-voltage towers, as well as the snow/dirt on them, are easily confused with iced cables. The complicated backgrounds can also distract the model. To solve this issue, the model needs to have scene understanding to grasp the layout of typical power transmission lines and to distinguish between the geometric features of the cables (slender and continuous) and towers (sturdy and angular). The model also needs to understand that ice should be attached to suspended cables rather than supporting structures, thereby accurately identifying the target lines.

This Ice-Seg dataset was adopted for image segmentation tasks. Both the comparative experiment and the ablation study demonstrate the superiority of our proposed U-ResNet on this dataset. Please review the detailed information in Section 3.

## Appendix C. Parameter Settings for ConvNeXt and InceptionNeXt

As mentioned in Section 3.6.3 and Section 3.7.3, although both the ConvNeXt and InceptionNeXt methods were designed for cross-paradigm tasks and have convolutional structures inspired by Swin Transformer, they require rough and different training environments and programming settings, which significantly increases difficulties in practice. We list their environments and settings below:

ConvNeXt for classification: Optimizer is AdamW rather than SGD for other methods (with initial learning rate 4 × 10^−3^, weight decay 0.05, β = (0.9, 0.999), eps = 1 × 10^−8^); learning rate scheduling is the cosine annealing strategy (decreasing from 4 × 10^−3^ to 1 × 10^−6^); five epochs of linear warmup (starting from 1 × 10^−6^); loss function is label smoothing cross-entropy (smoothing coefficient 0.1); and the regularization strategy is gradient clipping (max_norm = 1.0) with mixed precision training (FP16).

InceptionNeXt for classification: Use AdamW optimizer (learning rate 1 × 10^−3^, weight decay 0.01, β = (0.9, 0.999), eps = 1 × 10^−8^); learning rate scheduling is exponential warm-up for 10 epochs (starting from 1 × 10^−5^) followed by cosine annealing decay (learning rate decays from peak to 0 after warm-up); loss function is label smoothing cross-entropy (smoothing coefficient 0.1); and the regularization technique is gradient clipping (max_norm = 0.5), as well as mixed precision training (FP16).

ConvNeXt for segmentation: Use AdamW (learning rate 1 × 10^−4^, weight decay 0.01, β = (0.9, 0.999), eps = 1 × 10^−8^); learning rate scheduling is linear, warm-up for three epochs (starting from 1 × 10^−6^) first, then cosine annealing decay (decreasing from 1 × 10^−4^ to 1 × 10^−6^); the loss function combines the advantages of dice loss and focal loss and calculates positive sample weights based on the training set. Dynamic threshold optimization is used to search for the best segmentation threshold for the validation set every five epochs. Regularization techniques are gradient clipping (max_norm = 1.0) and mixed precision training.

Since InceptionNeXt was not designed for segmentation, we did not apply it in our segmentation experiments.
